# Utilization of institutional delivery service at Wukro and Butajera districts in the Northern and South Central Ethiopia

**DOI:** 10.1186/1471-2393-14-178

**Published:** 2014-05-28

**Authors:** Seifu Hagos, Debebe Shaweno, Meselech Assegid, Alemayehu Mekonnen, Mesganaw Fantahun Afework, Saifuddin Ahmed

**Affiliations:** 1 Department of Reproductive Health and Health Service Management, School of Public Health, College of Health Sciences, Addis Ababa University, Addis Ababa, Ethiopia; 2 School of Public and Environmental Health, College of Medicine and Health Sciences, Hawassa University, Hawassa, Ethiopia; 3 Bill & Melinda Gates Institute for Population and Reproductive Health, Population, Family and Reproductive Health, Johns Hopkins Bloomberg School of Public health, Baltimore, MD, USA

**Keywords:** Skilled attendance birth, Institutional delivery, Ethiopia

## Abstract

**Background:**

Ethiopia has one of the highest maternal mortality in the world. Institutional delivery is the key intervention in reducing maternal mortality and complications. However, the uptake of the service has remained low and the factors which contribute to this low uptake appear to vary widely. Our study aims to determine the magnitude and identify factors affecting delivery at health institution in two districts in Ethiopia.

**Methods:**

A community based cross sectional household survey was conducted from January to February 2012 in 12 randomly selected villages of Wukro and Butajera districts in the northern and south central parts of Ethiopia, respectively. Data were collected using a pretested questionnaire from 4949 women who delivered in the two years preceding the survey.

**Results:**

One in four women delivered the index child at a health facility. Among women who delivered at health facility, 16.1% deliveries were in government hospitals and 7.8% were in health centers. The factors that significantly affected institutional delivery in this study were district in which the women lived (AOR: 2.21, 95% CI: 1.28, 3.82), women age at interview (AOR: 1.96, 95% CI: 1.05, 3.62), women’s education (AOR: 3.53, 95% CI: 1.22, 10.20), wealth status (AOR: 16.82, 95% CI: 7.96, 35.54), women’s occupation (AOR: 1.50, 95% CI: 1.01, 2.24), antenatal care (4+) use (AOR: 1.77, 95% CI: 1.42, 2.20), and number of pregnancies (AOR: 0.25, 95% CI: 0.18,0.35). We found that women who were autonomous in decision making about place of delivery were less likely to deliver in health facility (AOR: 0.38, 95% CI: 0.23,0.63).

**Conclusions:**

Institutional delivery is still low in the Ethiopia. The most important factors that determine use of institutional delivery appear to be women education and household economic status.

Women’s autonomy in decision making on place of delivery did not improve health facility delivery in our study population.

Actions targeting the disadvantaged, improving quality of services and service availability in the area are likely to significantly increase institutional delivery.

## Background

The type of assistance a woman receives during childbirth has important health consequences for both the mother and child [1]. Since most maternal deaths and obstetric complications cluster around the time of delivery [2] and cannot be predicted a priori, skilled attendance at birth remains the most important intervention in reducing maternal mortality and complications. Skilled attendance during labor, delivery and the early post-partum period could reduce an estimated 13-33% of maternal deaths [3]. Owing to the central role of professional care at birth, skilled birth attendance was chosen as an indicator for monitoring progress towards the maternal health Millennium Development Goal-5 of reducing maternal mortality ratio by three quarters between 1990 and 2015 [4]. Earlier, the 1999 UN International Conference on Population and Development (ICPD + 5) declaration, set a goal of 50% of all births to be assisted by a skilled attendant by 2010, and 60% by 2015 in countries with very high maternal mortality. The corresponding global goals were 85% in 2010 and 90% in 2015 [5]. Recent data, however, suggest that the skilled attendance at birth (SBA) rate is very low in many settings, especially in sub-Saharan African and South Asian countries.

The World Health Organization (WHO) defines a skilled attendant as *“an accredited health professional-such as a midwife, doctor or nurse-who has been educated and trained to proficiency in the skills needed to manage normal (uncomplicated) pregnancies, childbirth, and the immediate postnatal period, and in the identification, management and referral of complications in women and newborns”*[6]. In this study we used institutional delivery (health facility delivery) to measure skilled attendance at birth because of the extreme rarity of skilled birth attendance at birth at home in Ethiopia and inability to decide whether a home delivery is a skilled delivery, according to the above definition.

Over the years, a number of studies have sought to identify factors that are associated with Institutional delivery. Many studies corroborated in reporting significant association with maternal education, household wealth, maternal age, autonomy of the mother and physical access to health institution [6-10]. We documented variation among studies is on the relative importance of reported factors. For instance, house hold wealth was recognized as more crucial determinant than access in India [7]. Similarly, though educational status of the mother was regarded as the most important factor in several other studies [7,8], the magnitude of its effect appears to be similar with that of access, household wealth and women’s autonomy in a study in Zambia [9]. Other determinants include quality of the service, previous or current antenatal care (ANC) follow up, previous health facility delivery and health care cost [7,10-13].

Recognizing the significance, the Federal Ministry of Health of Ethiopia (FMOH) identified low rate of institutional delivery rate as one priority area in the national Reproductive Health strategy and targeted to increase it to 60% by 2015 [14]. However, when only 3 years are left to conclude the plan period, only ten percent of births in Ethiopia are delivered at a health facility [1].

A review of in country studies indicates that factors associated with Institutional delivery appear to be context related and vary across ranges of studies not only in type but also in relative importance [1,12,15,16]. Unpredicted sudden onset of labour coupled with absence of transport facility or lack of money for transportation, lack of decision making by women, normal previous home deliveries are the major reasons cited for low institutional delivery in the country [12,16]. Studies in Ethiopia also reported that urban residence and maternal education are factors positively predicting institutional delivery [16,17]. However, studies were inconsistent in the association between ANC attendance during the index pregnancy and institutional delivery utilization [16,17]. Women who are informed that their pregnancy is normal (no risk) during ANC visits may be encouraged to give birth at home [11].

Hence it is important to understand contextual factors influencing institutional delivery in Ethiopia. Therefore, we conducted this study to examine the current status of institutional delivery rate and associated factors using a large data set in two districts of Ethiopia. The findings from this study will help the maternal and child health champions by providing factors operating in the context of Ethiopia.

## Methods

### Settings

This study was conducted in two districts namely Wukro and Butajera. Wukro district is located in eastern Zone, Tigray Regional state. The estimated size of the district is 2,068 km^2^ . The population of the district is estimated at 99,708 with the majority (95%) residing in rural areas. The vast majority of the district population (97%) follows the Orthodox Religion. Butajera district is located in *Guraghe* Zone, in the Southern Nations Nationalities and People’s Region (SNNPR). The estimated size of the district is 797 km^2^. The population of the district is estimated at 259,689 with the majority (87%) residing in rural areas. The vast majority of the district population (75%) follows the Islamic Religion. Farming is the main means of livelihood for the rural population of Butajera and Wukro districts. Each district is served by 2 hospitals and 2 to 3 health centers. In addition, there are health posts at the kebele level *(Kebele is the smallest administrative unit in Ethiopia).* Referral of patients may be made to a regional or zonal hospital between 50 and 100 kilometers away. According to Ethiopian Demographic and Health Survey (EDHS) 2011 report 11.6% and 6.1% of births occurred at health institutions in Tigray and SNNPR regions respectively. The study districts (Butajera and Wukro) were purposely selected because each district houses a health and demographic surveillance system (HDSS) (owned and operated by the two universities) with benefits of a better sampling frame and research infrastructure.

### Study design and period

The study employed a community based cross sectional study design. It was conducted from January to February 2012.

### Study population and sampling

The study population included all women who delivered within two years preceding the survey residing in the selected kebeles. The sample size was pre-determined by another objective but was adequate to answer this research question. The sample size was determined assuming skilled attendance at birth rate of 16% [18], 80% power and 95% confidence level, and a difference of 7% between those who were visited and not visited by Health Extension workers (HEWs), the calculated sample size was 1060 women who delivered in the two year preceding the survey. Because the study employed cluster sampling design for the survey, a design effect (deff) of 1.6 was considered for adjusting higher intracluster correlation in responses for sample size calculation. With an expectation of approximately 10% for non-response rate, a minimum of 1860 women who gave birth in last two years per district were required.

The study included a total 12 randomly selected villages (kebeles), of which 10 were rural and the remaining 2 were urban kebeles. The first phase of the data collection included mapping, enumeration of houses and household members to identify all women who had delivered in the two years preceding the survey and be used for developing sampling frame. The census resulted a total of 2296 and 2653 eligible households in Butajera and Wukro districts respectively. The study team subsequently decided to interview all eligible, consented women in the area to improve study power for other indicators and study objectives. This paper is hence based on 4949 women who were successfully interviewed.

### Data collection and instrument

Data were collected using a pretested questionnaire developed and adapted from EDHS and other published literatures. Data were collected on socio demographic characteristics such as place of residences, maternal age, level of education, religion, occupation, housing characteristics such as type of house, availability of certain household items, type of water supply and type of toilet, land size, obstetrics history such as place of delivery of last child (index), number of pregnancies, antenatal visits, number of deliveries, decision making on place of delivery, and reasons for choosing health facility for delivery.

Twenty female interviewers and two supervisors were recruited and trained in each of the two study districts. A five days training was given to the interviewers, supervisors, and coordinators on general techniques of interviewing and in the specific administration of each item in the questionnaire. A pretest was conducted in nearby districts which have similar basic socio-economic characteristics as the study districts.

### Data entry, processing and analysis

Data were double entered by trained data clerks. We used Stata (version 11, Stata Corporation, College Station, TX) for Data analysis.

Factor analysis (principal component analysis) was done using variables land size, type of house, availability of certain household items, type of water supply and type of toilet to construct “wealth status” [19].

The main study outcome is use of institutional delivery services (public or private hospitals and health centers deliveries) for the birth of the index (last) child. We identified the key candidates of the determinants of institutional delivery from literature review and conceptual frameworks on delivery service utilization in developing countries. These include maternal age, education, place of residence, marital status, occupation, religion, decision making on delivery place (autonomy), parity, gravidity and household economic status. We generated a variable “women autonomy” from the responses of the question on who decides on the place of delivery. The woman has autonomy if she decides on place of delivery by herself or together with her partner or other person. The woman who has no say in the decision is classified as women with no autonomy.

Descriptive analysis was done through summarization using percentages (frequency distribution), tables, graphs of the study variables and summary statistics.

We first checked the variables for multi collinearity by calculating variance inflation factor (VIF). We then applied complex survey data analysis command designating variables that contain information about the survey design, sampling unit (villages) and specifying the default method for variance estimation method. The variance was adjusted with Taylor linearized variance estimation method. No weighting was done (each observation is given a sampling weight of 1) as all eligible women who delivered within two years preceding the survey in the study kebeles were included. Multivariate logistic regression analysis adjusted for clustering due to cluster level sampling was then run to control for the effect of other factors. Odds ratios (95% confidence intervals) were calculated to determine the association between institutional delivery and predictor variables.

### Ethical clearance

The study protocol was approved by institutional review boards of Addis Ababa University College of health sciences and Johns Hopkins Bloomberg School of Public Health. Informed consent was received from the participants. Privacy and confidentiality of respondents was also maintained.

## Results

### Background

We included a total of 4949 women who delivered a child in the two years preceding the survey. This is higher than the calculated sample size of 3720 for both of the districts. No women declined from participation making the response rate 100.0%. However, there were an insignificant number of non-responses for certain questions.

The socio-demographic characteristic of the study population is shown in Table 1. Two thousand six hundred fifty three (53.6%) respondents were from Wukro District, Tigray Region, northern Ethiopia while 2296 (46.4%) were from Butajera, south central Ethiopia. Among the respondents, 3779 (76.4%) were rural residents and 1170 (23.6%) were urban residents. The mean (SD) age (year) of respondents is 28.6 (6.2) and ranges from 15-50 years. The age distribution indicates that a higher proportion of respondents 2527 (51.5%) are found in the age range 20-29. The great majority of the respondents were currently married (95.1%) and 63% percent of the respondents did not have any formal education. Occupationally, 2119 (42.8%) were housewives and 1951 (39.4%) combined housewife duties with farm work.

**Table 1:** Socio-demographic and fertility Characteristics of women who delivered a baby in the two years preceding the survey in Butajera and Wukro districts, 2012

**Characteristics**	**Number**	**Percent**
**District**		
Butajera	2296	46.4
Wukro	2653	53.6
**Mothers age (years)**		
15-19	224	4.5
20-29	2527	51.5
30-39	1898	38.3
40-49	291	5.9
Missing	9	0.2
**Place of residence**		
Urban	1170	23.6
Rural	3779	76.4
**Level of education**		
None	3098	62.6
Primary	1365	27.6
Secondary	402	8.1
College	84	1.7
**Marital status**		
Currently married	4706	95.1
Widowed, Divorced, never married	242	4.9
**Religion**		
Orthodox Christian	3281	66.3
Muslim	1412	28.5
Protestant	194	3.9
Catholic	59	1.2
Missing	2	0.04
**Occupation**		
Housewife	2119	42.8
Housewife and farm work	1951	39.4
Trade and other employee	596	12.0
Others	283	5.7
**Socio economic condition**		
Poorest	995	20.1
Poor	994	20.1
Middle	990	20.0
Rich	977	19.7
Richest	993	20.1
**Number of Pregnancies**		
1	959	19.4
2-4	2222	44.9
5-6	1026	20.8
7 and above	742	15.0
**Number of Deliveries**		
2	1775	35.9
4	1401	28.3
6	1026	20.8
7+	742	15.0
Missing	5	0.1

About 19% of the respondents were pregnant just once and 45% of the respondents had 2-4 pregnancies.

### Utilization of institutional delivery service

**Figure 1:**
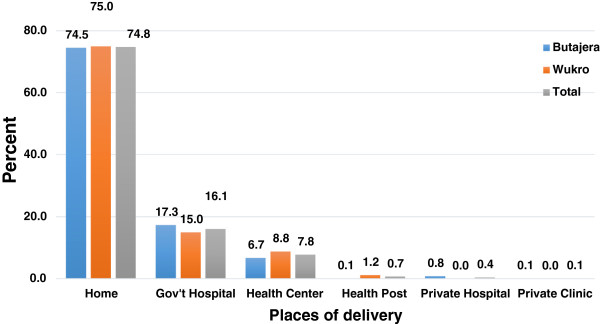
Percentage distribution of Places of delivery among women who gave birth in the previous two years in Butajera and Wukro districts, 2012.

Of the total respondents, 1,237 (25.0%) women delivered the index child at health facility in the two years preceding the survey, while the majority 3,712 (75.0%) delivered at home. Regarding health facility delivery, 793 (16.1%) delivered in government hospitals and 386 (7.8%) in health centers (Figure 1). Less than one percent of the deliveries took place in health posts and private clinics.

Table 2 shows the distribution of decision makers on place of delivery. Decisions were said to be mostly made by both the pregnant woman and husband/partner among 2361 (48%) respondents. Nearly equal number of respondents 2216 (44.9%) make a decision by themselves on place of delivery. It is only in less than one percent of the case that mother in laws or other relatives decide the place of delivery for the respondents (Table 2).

**Table 2:** Decision maker on place delivery among women who gave birth in the previous two years in Butajera and Wukro districts, 2012

**Decision maker for place of delivery**	**Number**	**Percent**
Respondent	2216	44.9
Husband/partner only	297	6.0
Both pregnant women and husband/partner	2361	47.8
Mother in law	25	0.5
Other relatives	30	0.6
TBA/TTBA^*^	4	0.6
HEW^**^	8	0.2
Missing	8	0.2

*Traditional/trained birth attendants, **Health Extension workers.

### Factors influencing institutional delivery

As shown in Table 3, bivariate analysis indicates that women place of residence, age at interview, educational status, wealth status, marital status, religion, occupation women autonomy, ANC visits, and number of pregnancies were significantly associated with institutional delivery.

**Table 3:** Association of socio-demographic characteristics, fertility and ANC visit with place of delivery in Butajera and Wukro districts, 2012

**Characteristics**	**Place of delivery**		**Crude OR (95% CI)**	**Adjusted OR (95% CI)**
	**Health facility (n,%)**	**Home (n,%)**		
**District**				
Butjira	574(25)	1722(75)	1.00	1.00
Wukro	663(25)	1990(75)	0.99(0.21, 4.59)	2.21(1.28,3.82)*
**Place of residence**				
Urban	704(60.2)	466(39.8)	1.00	1.00
Rural	533(14.1)	3246(85.9)	0.11(0.03,0.30)	0.55(0.29, 1.06)
**Mothers age (years)**^**#**^				
15–19	80(35.7)	144(64.3)	1.00	1.00
20–29	714(28.3)	1813(71.7)	0.70(0.55,0.91)	1.18(0.78,1.81)
30–39	408(21.5)	1490(78.5)	0.49(0.32,0.76)	2.03(1.22,3.38)*
40-49	32(11)	259(89)	0.22(0.14,0.35)	1.96(1.05,3.68)*
**Women’s education**				
None	440(14.2)	2658(85.8)	1.00	1.00
Primary	434(31.8)	931(68.2)	2.82(2.25,3.51)	1.20(0.99,1.46)
High school	291(72.4)	111(27.6)	15.8(8.76, 28.64)	2.54(1.65,3.90)*
College/University	72(85.7)	12(14.3)	36.2(15.4, 85.30)	3.53(1.22,10.20)*
**Wealth quintile**				
Poorest	86(8.6)	911(91.4)	1.00	1.00
Poor	98(9.9)	893(90.1)	1.62(1.09,2.41)	1.70(1.23,2.23)*
Middle	96(9.7)	892(90.1)	1.09(0.64,1.86)	1.60(0.98,2.63)
Rich	237(24.1)	745(75.9)	3.59(2.10,6.13)	4.11(2.39,7.06)*
Richest	720(72.7)	271 (27.3)	30.58(15.22, 61.43)	16.82(7.96,35.54)*
**Marital status**				
Currently married	1144(24.3)	3562(75.7)	1.00	1.00
Currently unmarried	93(38.3)	150(61.7)	1.93(1.48,2.52)	1.32(0.92,1.89)
**Religion**				
Orthodox Christian	829(25.3)	2452(74.7)	1.00	1.00
Muslim	315(22.3)	1097(77.7)	0.85(0.25,2.91)	0.79(0.65,0.95)*
Protestant	54(27.8)	140(72.2)	1.14(0.39,3.34)	0.88 (0.58,1.34)
Catholic	37(62.7)	22(37.3)	4.97(2.10,11.73)	1.10(0.76,1.60)
**Occupation**				
Farmer and housewife	235(12.1)	1716(87.9)	1.00	1.00
House wife	628(29.6)	1491(70.4)	3.08(1.50,6,31)	1.50(1.01,2.24)*
Employee	290(48.7)	306(51.3)	6.92(3.88,12.36)	1.64(1.08,2.50)*
Other	84(29.7)	199(70.3)	3.08(1.62,5.85)	1.25(0.79,1.99)
**Women Autonomy**^**##**^				
Not autonomous	155(41.7)	217(58.3)	1.00	1.00
Autonomous	1082(23.6)	3495(76.4)	0.43(0.24,0.77)	0.38(0.23,0.63)*
**ANC4 + visits**				
No	361(17.2)	1738(82.8)	1.00	1.00
Yes	876(30.7)	1974(69.3)	2.14(1.28, 3.55)	1.77(1.42, 2.20)*
**Number of pregnancies**				
1	474(49.4)	485(50.6)	1.00	1.00
2–4	532(23.9)	1690(76.1)	0.32(0.25,0.41)	0.39(0.30,0.5)*
5–6	158(15.4)	868(84.6)	0.18(0.13,0.26)	0.31(0.22,0.42)*
7 and above	73(9.8)	669(90.2)	0.11(0.07,0.17)	0.25(0.18,0.35)*
**Number of deliveries**				
1–2	722(40.7)	1053(59.3)	1.00	1.00**
3–4	282(20.1)	1119(79.9)	0.37(0.31,0.43)	
5–6	158(15.4)	868(84.6)	0.27(0.19,0.37)	
7 and above	73(9.8)	669(90.2)	0.16(0.10,0.24)	

*Significant associations (P < 0.05), **Not included in regression model because of high correlation with number of pregnancies.

#age at interview, ##Women autonomy on place of delivery.

In the multivariate logistic regression, we found that women living in Wukro district had two times (AOR = 2.21, 95% CI: 1.28-3.82) increased likelihood of delivery in health facility compared to Butajera. The odds of delivery in health facility among women 30 years old or above was about twice higher than those younger than 20 years old (AOR: 2.03; 95% CI: 1.22-3.38).

Women’s educational status was highly correlated with place of delivery. Those who had above secondary education had more than 2.5 times (AOR: 2.54; 95% CI: 1.65-3.90) higher chance of delivering in health facilities compared to the non-educated. Similarly women who had college education had more than 3 times (AOR: 3.53; 95% CI: 1.22-10.20) higher chance of delivering in health facilities compared to the non-educated. Women wealth status was found to be a strong predictor of health facility delivery. We found that women who are in the richest quintile had more than 16 times (AOR: 16.82; 95% CI: 7.96-35.54) chance of delivering in health facilities than those in the poorest quintile.

Women autonomy on deciding places for delivery was found to have an inverse relation with health facility delivery. We found that women who were autonomous (whose husband/partner or others decide) in decision making about place of delivery were less likely (AOR: 0.38, 95% CI: 0.23,0.63) to deliver in health facility than those who can’t decide by themselves.

Occupationally, the housewives and employees had a higher chance of giving birth in health facilities compared to those who combined household duties with farm work. Women who are housewives had more than 1.5 times (AOR: 1.50; 95% CI: 1.01-2.24) higher chance of delivering in health facilities compared to those who combined household duties with farm work. As the number of pregnancies increased, the chance of health facility delivery decreased significantly.

We also found a difference in health facility delivery by ANC visits during pregnancy. The chance of delivering in health facilities was almost more than twice among those who had attended ANC4 +.

However, marital status and place of residency did not show significant associations with health facility delivery.

### Reasons for choosing a particular facility for delivery

We further investigated the reasons for choosing a particular health facility for delivery service among 1237 women who delivered at health facility. The main reasons can be grouped into domains related to distance, quality and availability of services and perceived providers competence. Among the reasons, 589 (47.4%) women chose to deliver in a health facility because of its proximity to a home or work place. Service and medication availability are the next common reasons reported by 456 (36.9%) women. In addition, 462 (37.6%) of women reported availability of qualified professional, 454 (36.7%) provider’s knowledge and reputation and 428 (34.8%) cleanness of the facilities as the reasons for choosing facility delivery. One hundred eighty four (14.9%) women reported friendliness of providers (Table 4).

**Table 4:** Reasons for choosing health facility for delivery among women who delivered in the previous one year in Butajera and Wukro districts, 2012 (n = 1237)

**Reasons**	**Frequency**	**Percent of cases**
Close to my home/work	589	47.7
Required medication/service available only here/referred by provider	456	36.9
Doctor/health professional available	462	37.6
Provider knowledgeable/good reputation recommendation	454	36.7
Clean facilities	428	34.8
employer-designated site	240	19.5
Provider friendly/know the staff	184	14.9
Always come here	124	10.1
Had problem during delivery	48	3.9
can afford the fee	44	3.6
Baby overdue	21	1.7
Short waiting time	11	0.9
Had previous surgical delivery	6	0.5

## Discussions

In this study, we found that only a quarter of women delivered at health facility while the great majority delivered at home. We observed that most women decided on the place of delivery either by themselves or with their husband/partners. Closeness of health facility, medication and services availability, and providers’ reputation and perceived knowledge are the next most common reasons for choosing health facility for delivery. The factors that significantly influenced delivery at a health facility were district in which the women lived, women age at interview, wealth (economic) status, women’s occupation, Autonomy (decision making), ANC attendance, and number of pregnancies.

The finding that one in four women delivered in health facilities, although far from satisfactory, appears to be higher than reported (10%) in the recent Ethiopian Demographic Health Survey (EDHS) [1] and the recent report of the Federal Ministry of Health [20]. The reasons may need to be explored further but can be due to the HDSS activities in the studied districts. The two districts house HDSS sites where health and demographic surveillance activities, specific research and related communications might have influenced the use of health facilities for delivery. Although the timing of this research is not too far from that of EDHS 2011, rapid progress could have been made because of the attention that is now given by concerned stakeholders as the country lags behind in the indicator.

We found that women living in Wukro district had a higher likelihood having a health facility delivery as compared to women in Butajera. We observed the following important difference in maternal health services delivery between the districts. In Wukro, we observed that the district has made available ambulance services for laboring mothers in the rural area. Moreover, Health Extension Workers (HEWs) residing in the villages facilitate facility delivery by calling ambulance for laboring mothers. On top of these, the Traditional Birth Attendants (TBAs) in the district are trained and acting as promoter of facility delivery. These differences might have resulted in a higher likelihood of having a health facility delivery among women living in Wukro district.

Unlike other studies, we didn’t observe a difference in the use of health facility for delivery among rural and urban women. However, many studies consistently indicate that rural residents had a lower chance of institutional delivery [7,8,12,21-26]. The most common reason reported for choosing health facility for delivery was being close to home. Distance to health facility is a common reason for not delivering in health facilities [1,8,12,22,25-27]. Women in the rural setting are disadvantaged in terms of access to services (distance) and other determinants. Improving physical access to delivery services, availing the required professional and necessary supplies for the rural women would be important to narrow the gap.

In the multivariate analysis, older women had a higher chance of delivery in health facilities than their younger counterparts. The relations between age and institutional delivery in the literature have conflicting findings. Studies have reported that older women may believe that there is less risk to home delivery due to previous uneventful pregnancies and deliveries [12]. On the other hand, older women may become knowledgeable during successive ANC visits on the benefits of health facility deliveries. Obstetric complications may increases with age as a result older women may use health facility for delivery than younger women [11]. Hence ANC visits for younger mothers should focus on the benefits of institutional delivery as well as the unpredictability of risks and complications during the course of pregnancy and delivery.

The finding that ANC attendance is associated with institution delivery is in line with the expectation and is also reported by other studies [1,28,29]. A study in Kenya reported that the use of institutional delivery services was very low even among antenatal care attendees. The study concluded that since women with ANC visits have already demonstrated some acceptance of the healthcare system presenting a readily-accessible opportunity for one-on-one counseling on the benefits of delivering at a health facility. The healthcare providers should take the full advantage of this opportunity [25]. Nonetheless, some authors argue that ANC would have an inverse association with delivering at health facility as women who are told their pregnancy is fine may feel encouraged to deliver without any professional help [11]. The fact that many women who attended ANC were not delivering in health facilities can be considered as missed opportunity. In our study, among 2,850 women attending ANC4+, only 1974 (30%) delivered at health facilities. On top of the reasons mentioned above, improving the quality of ANC services such as how it is tailored to the need of each pregnant woman might help to improve service uptake. It is equally important to know about those who might have come, but did not get the services for any reasons related to the facility.

We found that women’s education level specifically high school and college education increased the likelihood of facility delivery. Women education is reported to have a strong and dose dependent positive effect on the use delivery services [9]. Women who are educated might have access to information, better knowledge on services, access and control over resources and thus might better use health facility for delivery.

Women who decide on place of delivery by themselves are less likely to deliver at health facility than women whose husband/partner or others decide. A review literature on the determinants of delivery service use indicates the significant role and influence of women autonomy and status on the use delivery care [11]. Our finding indicates that women are the ones who chose to deliver at homes while their husbands might have chosen delivery at a health facility. Moreover, women in the districts have a relatively high chance to decide where to deliver but they prefer to deliver at home. A possible reason is that - the health facility environment may not be appealing to the women which includes health worker’s approach, long waiting time, not allowing relatives to be present in the delivery room. This finding has programmatic implication. Ensuring women autonomy in deciding place of delivery alone may not guarantee maternal health service utilization. Improvement in health facility delivery may require better awareness creation on the benefits of facility delivery, and creation of conducive health service delivery environment.

A much larger difference was noted in place of delivery by wealth status. Those in the richest quintile had about 16 times the chance of delivering in health facilities. A study in eleven administrative regions of Ethiopia reported that distance and cost were major constraints in using health facilities for delivery [30]. Many studies showed the importance of women’s education and particularly wealth in maternal health service utilization [3,10,25,31-42]. Hidden costs such as transportation cost, unofficial provider fees and other opportunity cost for the mother and accompanying person(s) deter particular poor families from using delivery services [11]. In Ethiopia, maternal health services are given free of charge by law [43], however, there is policy practice gap in implementation of the law [44]. Hence, adaptation and implementation of targeted interventions for the poor women is necessary. Examples of experiences of other countries include cash assistance in Guinea, Mauritania and Burkinafaso [45], pro-poor fee exemption in Ghana, Senegal and Tanzania [45-49] and implementation of programmes targeted to the poorest women through subsidizing emergency obstetric and neonatal care (in Bruknafaso [50,51], conditional cash transfer in India [52] and voucher and equity funds in Cambodia [45,53] where poorest women were identified by health care workers through home to home visit and social health insurance in Bolivia [45].

Occupationally, the housewives had a higher chance of giving birth in health facilities compared to those who combined household duties with farm work. A possible explanation is that those that combine household work with farming are engaged in many activities than housewives. These women would be relatively very busy and might spent most of their time on farm and domestic activities.

As the number of pregnancies/deliveries increased the chance of health facility delivery decreased significantly. This is in line with findings of the 2011 EDHS [1]. Possible explanations are women who have large number of children would spend time caring for the children. In addition, previous uneventful pregnancies and deliveries may lead to undermine risks for poor outcomes.

In summary, findings of this study on factors associated with use of health facility for delivery concur with some and differ from other studies. As a matter of fact, review of literature on factors related to institutional delivery is filled with numerous contradicting findings as presented above and elucidated by systematic review of literature in developing countries which emphasized the need to thoroughly explore and address context-specific causes [40]. Methodological differences explain part of the differences in the findings of these studies [11]. These differences are related with designs, sampling techniques, standardization of inclusion and exclusion criteria, analysis methods (some use multilevel modeling, some adjust for clustering and other do not, selection of exposure variables in the model) and contextual difference (for example definition of distance can vary between countries),

### Limitations of the study

The findings of this study should be interpreted with the following limitations. The cross sectional nature of the study does not allow establishing causality of associations and the results should be interpreted cautiously. Recall bias cannot be ruled out about events that took place further from the period of data collection, although training of data collectors, use of local events and provision of manuals for interviewing and the unforgettable nature of the major events play a considerable role in minimizing biases.

As the study districts are purposively selected, the findings of this study can only be generalized to settings with similar characteristics.

Despite the above limitations, the design of the study (being a community based) and the relatively larger sample size used has given the study adequate power.

## Conclusions

Although far from satisfactory, the institutional delivery rate in this study is higher than the national average documented. Factors such as maternal age, occupation, economic status, ANC attendance showed a positively influence on institutional delivery. The findings of this study showed the importance of context in the influence of the different factors that affect institutional delivery services.

Actions targeting the disadvantaged (poor, rural residents, and illiterate) and encouraging the use ANC services is expected to improve institutional delivery in the study areas. Since perceived quality of service appears to be a reason for choice of health facility, improving the quality of service through making available health professionals and necessary medications, improving cleanliness of facilities and provision of mothers’ friendly services can improve the utilization of delivery service.

## Competing interests

The authors declare that they have no competing interests.

## Authors’ contributions

MF designed and lead the study. AS helped in the analysis of the data and drafting the manuscript. SH, MA, DS, and AM coordinated the data collection and management. All authors participated in the data analysis and drafting of the manuscript. All authors read and approved the final manuscript.

## Pre-publication history

The pre-publication history for this paper can be accessed here:

http://www.biomedcentral.com/1471-2393/14/178/prepub
